# Investigating the effects of age and conditioning stimulation intensity on SMA–M1 connectivity in younger, middle-aged, and older adults

**DOI:** 10.1007/s00421-025-05904-0

**Published:** 2025-07-21

**Authors:** Jane Tan, Grant Rowe, Rohan Puri, Merrilee Needham, Michelle Marneweck, Shivani Radia, Ann-Maree Vallence

**Affiliations:** 1 Discipline of Psychology, College of Science, Health, Engineering and Education, Murdoch University, 90 South Street, Murdoch, Perth, WA 6150 Australia; 2 Centre for Healthy Ageing, Health Futures Institute, Murdoch University, Perth, WA Australia; 3 Sensorimotor Neuroscience and Ageing Research Group, School of Psychological Sciences, College of Health and Medicine, University of Tasmania, Hobart, TAS Australia; 4 Centre for Molecular Medicine and Innovative Therapeutics, Murdoch University, Perth, WA Australia; 5 Fiona Stanley Hospital, Perth, WA Australia; 6 Perron Institute for Neurological and Translational Science, Perth, WA Australia; 7 South Metropolitan Health Service, Perth, WA Australia; 8 Department of Human Physiology, University of Oregon, Eugene, OR USA

**Keywords:** Bilateral motor control, Supplementary motor area, Primary motor cortex, Transcranial magnetic stimulation, Functional connectivity, Ageing

## Abstract

**Purpose:**

This study aimed to investigate bilateral motor control and connectivity between supplementary motor area (SMA) and primary motor cortex (M1) in younger, middle-aged, and older healthy adults.

**Methods:**

32 younger (mean age 22.8 ± 5.3 years), 18 middle-aged (47.6 ± 6.5 years), and 23 older (75.8 ± 6.7 years) adults were tested. Bilateral motor control was assessed using the Purdue pegboard. Dual-site transcranial magnetic stimulation (TMS) was used to measure SMA–M1 connectivity at different conditioning stimulation intensities.

**Results:**

Older adults had significantly poorer motor performance than younger and middle-aged in all pegboard subtests. Notably, there were no conclusive differences in motor performance between younger and middle-aged adults. There was no conclusive evidence supporting age-related and intensity-related differences in SMA–M1 connectivity between younger, middle-aged, and older adults. There was also no conclusive evidence to support clear associations between SMA–M1 connectivity and bilateral motor control.

**Conclusion:**

Age-related declines in bilateral motor functioning was found in older, but not middle-aged adults. The bilateral motor functioning of middle-aged adults is more young-like than old-like. The lack of conclusive age- and intensity-related differences in SMA–M1 connectivity, and lack of conclusive association with bilateral motor performance, might be due to high inter-individual variability in SMA–M1 connectivity. Potential factors contributing to this variability include SMA and M1 morphometry, the structural connectivity between these regions, and the localisation of SMA.

**Supplementary Information:**

The online version contains supplementary material available at 10.1007/s00421-025-05904-0.

## Introduction

Bilateral motor control is crucial for many fundamental daily activities, such as eating and dressing (Maes et al. 2017). While declines in bilateral motor control can be observed from middle age (Vieluf et al. 2015), age-related changes in motor control have largely been studied in older adults as these decrements are detrimental to their functional independence and reduce their quality of life (Krehbiel et al. 2017).

The supplementary motor area (SMA), a midline cortical region, is involved in bimanual movement preparation and execution (Lara et al. 2018; Malouin et al. 2003; Nachev et al. 2008; Welniarz et al. 2019) and has direct cortical projections to the primary motor cortex (M1) (Dum and Strick 2002; Luppino et al. 1993). A dual-coil paired-pulse measure of connectivity (henceforth referred to as connectivity) between SMA and M1 can be measured using transcranial magnetic stimulation (TMS) to determine the degree to which SMA activity causally affects M1 activity (Arai et al. 2011, 2012; Friston 2011; Hallett et al. 2017). Specifically, a suprathreshold intensity conditioning stimulus (CS) is delivered to SMA before a test stimulus (TS) is delivered to M1 at interstimulus intervals (ISI) of 6–8 ms. This results in a larger motor evoked potential (MEP) amplitude than when TS to M1 is delivered alone, suggesting that SMA exerts a faciliatory effect on M1 (Arai et al. 2011, 2012). While the mechanisms underlying SMA–M1 facilitation have not been directly explored in humans, research in primates suggest that SMA–M1 facilitation is likely due to glutamatergic N-methyl-D-aspartate (NMDA) receptors and non-NMDA receptors (Shima and Tanji 1998).

Given the role of SMA in bilateral movement, and the well-characterised age-related decline in bilateral movement control, previous research has investigated SMA–M1 facilitation at a single CS intensity in younger and older adults. SMA–M1 facilitation using a CS intensity of 140% of active motor threshold (AMT) was significantly reduced in healthy older adults when compared with younger adults (Green et al. 2018; Rurak et al. 2021b). Importantly, greater SMA–M1 facilitation using this CS intensity was significantly associated with better bimanual motor performance in healthy older individuals, and not in younger individuals (Rurak et al. 2021b). This suggests that age-related differences in SMA–M1 connectivity might underpin, in part, age-related declines in bilateral motor control. However, SMA–M1 connectivity has not been investigated in middle-aged individuals, a cohort that is frequently overlooked in research (Lachman 2015). Existing research indicates that middle-aged adults performed significantly better than older adults on fine motor tasks (Hamilton et al. 2017), and declines in fine motor skills were significantly associated with increasing age in a large sample of individuals aged 45 years and older (Hoogendam et al. 2014). At present, there is a lack of research investigating the neural underpinnings of bilateral motor control in middle-aged individuals. This is a research gap that should be addressed to achieve a better understanding of the neurophysiological changes that occur between young and older adulthood (Lachman 2015). This can help to identify any neurophysiological changes in midlife that could be predictive of subsequent changes in motor control in later life, which can aid in the development of preventative or early interventions (Lachman 2015).

Thus, the current study aimed to investigate SMA–M1 connectivity across the lifespan: specifically, in younger (18–39 years), middle-aged (40–59 years), and older (≥ 60 years) adults. SMA–M1 connectivity was investigated over a range of CS intensities—CS intensity has been shown to have significant and non-linear effects on intracortical activity within M1 (Peurala et al. 2008) and cortico-cortical activity between other brain regions (Koch et al. 2007), such that higher CS intensities are not necessarily associated with higher cortical activity. A systematic examination of the effect of CS intensity on SMA–M1 connectivity might provide valuable insight into putative mechanisms subserving this facilitatory pathway. Bilateral motor performance was also measured in younger, middle-aged, and older adults to investigate age-related differences in motor functioning, and age-related differences in the association between SMA–M1 connectivity and bilateral motor control.

In view of previous literature (Green et al. 2018; Rurak et al. 2021b), we hypothesised that SMA–M1 facilitation would be lower for older adults compared to middle-aged and younger adults, and lower for middle-aged adults compared to younger adults. We further hypothesised that CS intensities would have significant and non-linear effects on SMA–M1 connectivity, with highest SMA–M1 facilitation when CS intensity is 140% of AMT (Koch et al. 2007). We also hypothesised that greater SMA–M1 facilitation will be significantly associated with better bilateral motor performance for middle-aged and older adults, but not for younger adults (Rurak et al. 2021b).

## Methods

### Participants

A total of 92 healthy adults were recruited in the study. However, 19 participants were withdrawn during data collection due to two technical reasons: (1) the defined SMA and M1 sites were too close to position TMS coils simultaneously (*n* = 11), and (2) the dual-site CS intensity at 140% of AMT was greater than the maximum stimulator output (MSO) (*n* = 8). Therefore, 73 self-declared right-hand dominant adults participated in the current study and were categorised into three age groups (younger [18–39 y]: *n* = 32, 22.8 ± 5.3; middle-aged [40–59 y]: *n* = 18, 47.6 ± 6.5; older [≥ 60 y]: *n* = 23, 75.8 ± 6.7). The participants were recruited from the local community. All participants were screened for TMS (Rossi et al. 2011, 2021) and magnetic resonance imaging (MRI) contraindications. The Murdoch University Research Ethics Committee approved the study (2019/033), and all participants provided written informed consent.

### TMS

Electromyographic (EMG) activity was recorded using Ag-AgCI surface electrodes placed in a belly-tendon montage on the right first dorsal interosseous (FDI). The EMG signal was amplified 1000 × and bandpass filtered at 20–1000 Hz (Cambridge Electronic Design (CED) 1902 signal conditioner, Cambridge, UK) before been digitised at a sampling rate of 5 kHz (CED 1401 analog-to-digital converter, Cambridge, UK).

#### M1 stimulation

A figure-of-eight coil (50 mm diameter) connected to a MagStim 200^2^ stimulator (Magstim Co., Whitland, Dyfed, UK) was used to stimulate the left M1 (posterior-anterior current induced) at the location that generated the largest and most consistent MEPs. The M1 stimulation intensity was set as the percentage of MSO that elicited peak-to-peak MEP amplitudes of ~ 1 mV in the resting FDI (SI_1mV_).

#### SMA stimulation

A second figure-of-eight coil (50 mm diameter) connected to another MagStim 200^2^ stimulator was used to stimulate the left SMA (current directed from the midline towards SMA). The left SMA was targeted as the incidence and magnitude of SMA–M1 facilitation was found to be greater when the stimulated SMA is ipsilateral to the stimulated M1, than contralateral SMA (Côté et al. 2020). To identify the SMA anatomically, a T1-weighted image from a 3.0 T MR scanner (Siemens Magnetom, Erlangen, Germany) was uploaded to neuronavigation software (BrainSight, BrainBox, Cardiff, UK) to reconstruct skin and brain curvilinear images. The anterior commissure (AC) and posterior commissure (PC) were defined in native subject space by manually identifying the landmarks on each participant’s T1-weighted MRI. A straight line was then positioned through the AC and PC to generate the AC–PC line. Landmarks were identified by visually inspecting the mid-sagittal slice, with the AC located as a compact white matter bundle at the anterior wall of the third ventricle, and the PC as a distinct tract at the dorsal midbrain, just above the cerebral aqueduct (Gaillard, (Gaillard 2018)) (see Fig. S1 in Supplementary Materials). The anterior border of the anatomical SMA was determined by positioning a perpendicular line to the AC–PC line at the anterior commissure and marking its intersection with the cerebrum surface. The anatomical SMA area was subsequently defined from the anterior border to the precentral sulcus (HiroShima et al. 2014; Luders 2008; Picard and Strick 1996; Potgieser et al. 2014; Vorobiev et al. 1998). If the SMA and MI sites were too close to allow both coils to be placed on the scalp, the site used for SMA stimulation was 4 cm anterior of Cz (Cz + 4) (International 10–20 System), consistent with previous research (Arai et al. 2011, 2012). There were 37 participants who were tested at the anatomical SMA site (younger: 14, middle-aged: 12, older: 11) and 36 participants tested at the Cz + 4 site (younger: 18, middle-aged: 6, older: 12).

### Experimental protocol

Participants obtained an anatomical MRI at a local radiology facility prior to the TMS session (MRI and TMS session were completed on separate days). SMA–M1 connectivity was assessed using SI_1mV_-alone stimuli and dual-site stimuli targeting SMA and M1. During SI_1mV_-alone trials, a single SI_1mV_ pulse was delivered to M1. During dual-site trials, a CS was delivered to SMA followed by a TS delivered to M1 (SI_1mV_) with an ISI of 7 ms (Rurak et al. 2021a, 2021b). This was based on a previous study that found reliability of SMA–M1 connectivity to be highest at an ISI of 7 ms compared to 6 or 8 ms (Rurak et al. 2021a). Dual-site TMS was delivered with six CS intensities: 100, 110, 120, 130, 140 and 150% of AMT. AMT was determined with the participant performing an isometric abduction contraction of the right index finger against a fixed block at 10% of maximum muscle activation (root mean squared [RMS] EMG signal was provided as visual feedback). AMT was defined as the minimum TMS intensity (as a percentage of MSO) that elicited MEPs in the FDI of at least 0.2 mV from at least 3 out of 5 consecutive trials (Rossini et al. 2015). Therefore, there were seven TMS types (TMS TYPE): SI_1mV_-alone and six dual-site. Participants received TMS in blocks of 28 trials: 4 trials of each TMS TYPE per block. A total of 7 blocks were delivered in each session, resulting in a total of 28 trials per TMS TYPE. Trials were pseudo-randomised within the blocks at an intertrial interval of 5 s (± 10%).

### Motor function

Motor function was assessed using the Purdue pegboard (Lafayette Instrument, USA). Participants were instructed to complete 4 subtests in a fixed order: unimanual with the right and left hands, simple bimanual, and assembly. The fixed sequence was selected as it is consistent with standard Purdue Pegboard administration protocols (Tiffin and Asher 1948). For all subtests, participants were instructed to complete the tasks as quickly as possible. For the unimanual subtests, participants were instructed to pick up pegs and place them in wells set out in columns on the pegboard. The total number of pegs placed successfully in the wells within 30 s was scored. For the simple bimanual subtest, the participants used both hands to simultaneously pick up pegs and place them in the wells in the corresponding column. The total number of rows (i.e., pairs) with pegs successfully placed in the wells in 30 s was scored. For the assembly subtest, the participants had to alternate between hands (starting with their right hand) picking up and placing items to build a four-item assembly. The total number of items assembled within 60 s was scored.

### Data processing

All SI_1mV_-alone and dual-site TMS TYPEs were captured with Signal software (CED, Cambridge, UK). The trials were processed with a customised script to calculate peak-to-peak MEP amplitudes (from 10 to 60 ms after the TS) as well as the root mean square (RMS) of background EMG activity during the 100 ms preceding TMS. This was used to identify and exclude trials with elevated muscle activity, defined as those exceeding the session’s mean RMS by more than two standard deviations. For 10 participants, the dual-site TMS TYPE at 150% of AMT was not undertaken because the calculated intensities were greater than the MSO. The coefficient of variation (CV) of the SI_1mV_-alone trials was determined for each participant to investigate whether the variability in the single-pulse MEP amplitude influenced the SMA–M1 ratio results.

### Statistical analysis

Trial-level MEP amplitudes were analysed with a Bayesian generalised linear mixed model (GLMM) using a gamma distribution and log link function, with fixed effects of AGE (younger, middle-aged, older), TMS TYPE (SI_1mV_-alone, dual-site: 100, 110, 120, 130, 140, and 150% of AMT) and SITE (anatomical SMA, Cz + 4). Trial-level data are increasingly analysed using GLMMs in TMS research, as this approach accommodates the inherent variability in responses both within and between participants (Brown 2021; Opie et al. 2020; Puri and Hinder 2022; Stange 2024; West et al. 2022). Each trial influences the model predictions, providing a more nuanced understanding of the data. This approach leverages mixed-effects models to capture both fixed and random effects, enhancing statistical power and sensitivity while reducing bias from outliers.

Differences in AMT between AGE were examined with a Bayesian GLMM using a beta distribution, where AMT values were scaled to a 0–1 interval by dividing them by 100.

Differences in CV of SI_1mV_-alone trials between AGE and SITE were examined with a Bayesian GLMM using a gamma distribution and log link function.

Motor function was assessed with a Bayesian GLMM with AGE and SUBTEST (right hand, left hand, simple bimanual, assembly) fixed effects using a Poisson distribution and log link function.

For the MEP amplitude and motor function models, the posterior distributions were generated by running four independent chains, each with 1500 warm up and 4500 post-warm up samples (18,000 total post-warm up samples) using the NUTS extension of Hamiltonian Monte Carlo. For the AMT model, the posterior distribution was generated by running four independent chains, each with 5000 warm up and 5000 post-warm up samples (20,000 total post-warm up samples). For the CV of SI_1mV_-alone model, the posterior distribution was generated by running four independent chains, each with 8000 warm up and 12,000 post-warm up samples (48,000 total post-warm up samples). The population-level parameters were assigned improper flat priors over real values (Puri et al. 2023; Puri and Hinder 2022). Flat priors were deliberately chosen to allow the data to primarily drive the posterior estimates, avoiding bias from prior assumptions. This approach aligns with traditional frequentist inference, where prior information is not incorporated. Given the limited prior research on this specific question, we believe that more foundational work is needed before introducing informative priors in future analyses. Additionally, the maximal random effect structure (i.e., by-participant random intercepts and by-participant random slopes for all fixed effects as well as correlations among random effects) as allowed by the data and justified by the design was specified for every model (Bari and Robbins 2013). After model fitting, specific comparisons were conducted to analyse main and interaction effects, except for the CV of SI_1mV_-alone model where the additive effects of AGE and SITE were examined. The posterior distribution of the comparison was used to derive indicators of effect existence (i.e., the consistency of an effect) and significance (i.e., the magnitude of the effect) (Makowski et al. 2019; Puri et al. 2023; Puri and Hinder 2022). To assess effect existence, the probability of direction (pd) was calculated, which is the percentage of the posterior distribution aligned with the median’s sign, ranging from 50% (least consistent) to 100% (most consistent). The pd correlates closely with frequentist p-values; for example, a pd of 97.5% corresponds to a two-sided p-value of 0.05 (Makowski et al. 2019). A region of practical equivalence (ROPE) was defined as a range of values (± 5%) around the null value for contrasts, i.e., [0.95, 1.05], centred on 1.00 as differences are ratios on the response scale. This threshold reflects a ± 5% region around “no difference” that is practically equivalent (i.e., too small to be of practical significance) and was chosen to indicate that changes greater or lesser than this are considered practically meaningful. Following this, an 89% highest density interval (HDI) was established, indicating the range within which 89% of the posterior distribution lies. Then, using both the HDI and ROPE, the percentage of HDI falling within the ROPE (% of HDI in ROPE) is reported and the following decision rule is applied (Kruschke 2018): if the HDI entirely falls within the ROPE, the null hypothesis of no effect (H_0_) is accepted; if it completely falls outside the ROPE, H_0_ is rejected; if it partially overlaps, no decision is made regarding H_0_.

Model diagnostics were thoroughly evaluated. All potential scale reduction factor (R̂) values were 1.00—except for one group-level variance term with R̂ = 1.01—indicating excellent convergence across parameters. Both Bulk and Tail effective sample sizes were consistently high, confirming the reliability of the posterior estimates. Minor divergent transitions were detected in two models: three in the verified SMA-proper model (filtered_tms_model: Gamma family, N = 12,269) and one in the active motor threshold model (mt_model_beta: Beta family, N = 73) (see Supplementary Materials). These accounted for less than 0.02% of total post-warmup draws and were not associated with any unstable parameter behaviour or concerning diagnostics. Posterior predictive checks also showed strong agreement between predicted and observed distributions, further supporting the adequacy of the model fit.

To assess the association between SMA–M1 connectivity and assembly pegboard performance across age groups, as well as the relationship between MEP variability and SMA–M1 connectivity (collapsed across age groups) at different stimulation sites, we employed a Bayesian version of Spearman’s rank correlation. This approach involves rank-transforming both variables to account for potential non-normality and non-linearity, followed by computation of a Bayes factor correlation on the ranks. Posterior samples were drawn and summarised using an 89% highest density interval (HDI) and a region of practical equivalence (ROPE) of ± 0.10. This narrower ROPE reflects a conservative threshold for detecting practically meaningful correlations, consistent with conventions where |r|< 0.10 is considered negligible (Schober et al. 2018).

Statistical analyses and data visualisation were performed via the software package R for Statistical Computing version 4.4.1 (R Core Team 2024) using RStudio IDE version 2024.04.2 + 764 and packages ‘bayestestR’ (Makowski et al. 2019), ‘brms’ (Bürkner 2021), ‘cowplot’ (Wilke 2025), ‘emmeans’ (Lenth 2025), ‘ggpubr’ (Kassambara 2025), ‘ggsignif’ (Ahlmann-Eltze and Patil  2021), ‘insight’ (Lüdecke et al. 2024), ‘skimr’ (Waring et al.  2025), and ‘tidyverse’ (Wickham  2019).

## Results

### Purdue pegboard performance

**Fig. 1:**
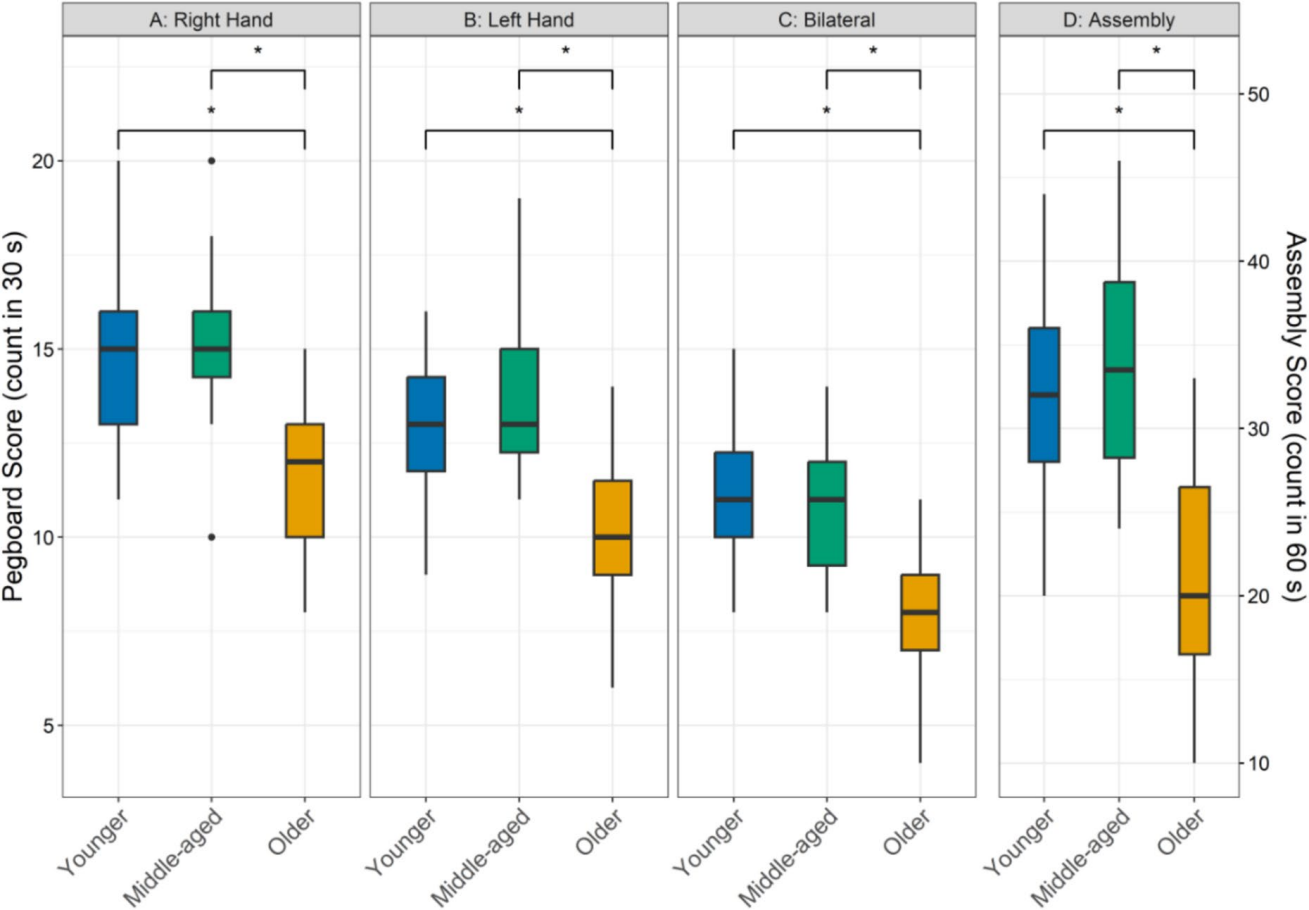
Panels **A** and **B** show pegboard performance for the unilateral subtests (Right Hand and Left Hand), Panel **C** shows performance for the bilateral subtest, and Panel **D** shows performance for the assembly subtest for younger (18–39 years; blue bars), middle-aged (40–59 years; green bars), older adults (60 + years; yellow bars). The lower and upper hinges represent the 25th and 75th percentiles, respectively. The whiskers extend to values that are up to 1.5 times the interquartile range (the difference between the 25th and 75th percentiles) below the lower hinge and above the upper hinge. Points that lie beyond the whiskers are shown as filled circles, indicating outliers. An asterisk indicates a practically significant result

**Fig. 2:**
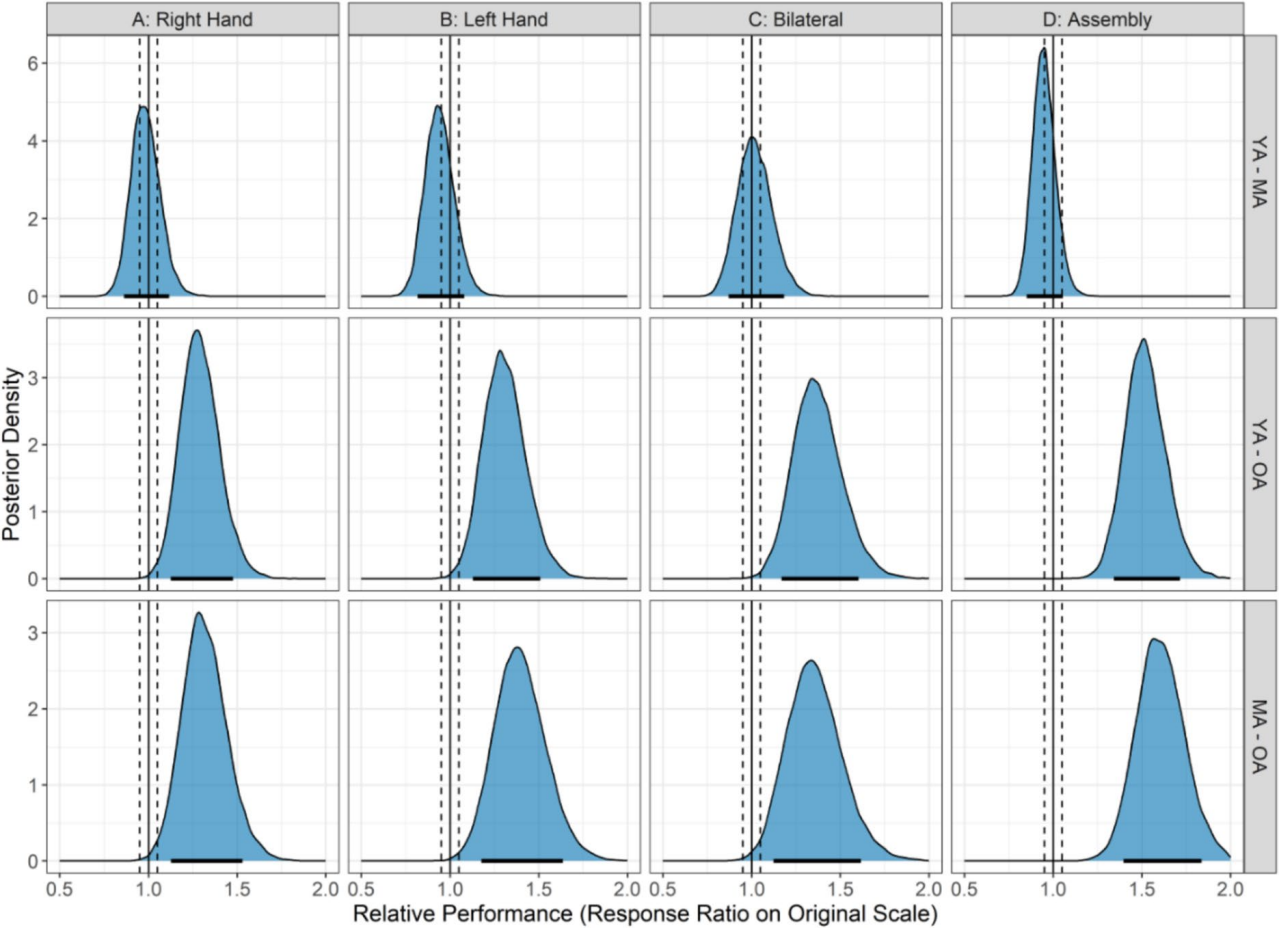
Posterior distributions of pairwise contrasts for pegboard performance across each subtest. The x-axis displays response ratios on the original performance scale, where 1.0 (solid black line) indicates no difference between groups; the y-axis represents the relative likelihood of each value. Vertical dashed lines denote the Region of Practical Equivalence (ROPE: 0.95–1.05). Black bars indicate the 89% Highest Density Interval (HDI); when the HDI lies entirely outside the ROPE, it suggests a practically meaningful group difference. HDIs positioned to the right of 1.0 indicate better performance in the numerator group relative to the denominator. YA, MA, and OA refer to younger, middle-aged, and older adults, respectively

Figure 1 shows performance on the four subtests of the Purdue Pegboard for the three age groups. Analyses showed that older adults performed worse than younger adults (28.5–51.5% difference; all pds > 99.8%; 0% in ROPE) and middle-aged adults (31.0–60.2%; all pds > 99.6%; 0% in ROPE) across all pegboard subtests. There were inconclusive differences between younger and middle-aged groups for all subtests (1.4–6.2%; all pds < 78.9%; all HDIs in ROPE between 35.3 and 48.1%). Figure 2 shows the posterior distributions of all pairwise group contrasts for pegboard performance across each subtest.

### Effect of conditioning stimulation intensity on SMA–M1 connectivity across different age groups

#### Corticospinal excitability

The AMT (mean ± *SD*) for the age groups were as follows: younger, 48.5% ± 8.9; middle-aged, 51.9% ± 10.0; and older, 59.3% ± 10.3. The AMT analysis revealed meaningful age-related differences. AMT was greater for older adults than younger adults (35.1% higher (95% CI 20–47%); pd = 100.0%; ROPE = 0.0%) and middle-aged adults (26.0% higher (95% CI 5–42%); pd = 100.0%; ROPE = 0.0%). The difference in AMT between younger and middle-aged adults was inconclusive (pd = 100.0%; ROPE = 19.1%).

Analysis of single-pulse MEP amplitude revealed inconclusive evidence for a two-way interaction between AGE group and SITE. The pd values for the interaction contrasts ranged from 64.3 to 78.5%, and all posterior distributions showed a high percentage within the ROPE (6.9–10.4%), suggesting that the magnitude of these differences was inconclusive. Main effects of AGE and SITE also showed limited evidence of systematic differences. The AGE contrasts yielded pd values between 61.6 and 82.6%, with ROPE percentages between 12.7 and 17.9%, while the SITE contrast had a pd of 83.6 and 14.4% of its posterior within the ROPE. These findings indicate inconclusive effects of AGE and SITE on single-pulse amplitude. All posterior distributions were symmetric with light tails, confirming the absence of strong skew or extreme values. This reinforces the stability of central tendency estimates and suggests that the reported effects were not driven by outliers or distributional irregularities.

The percentage of trials excluded due to excessive EMG background noise (mean ± *SD*) was 2.6% ± 1.9 for younger adults, 2.5% ± 1.9 for middle-aged adults, and 3.1% ± 2.1 for older adults. For all age group comparisons, the percentage of the posterior within the ROPE (± 0.5%) ranged from 39 to 64%, indicating that the differences in excluded trials are inconclusive and do not provide strong evidence for meaningful differences between the groups.

#### SMA–M1 connectivity

**Fig. 3:**
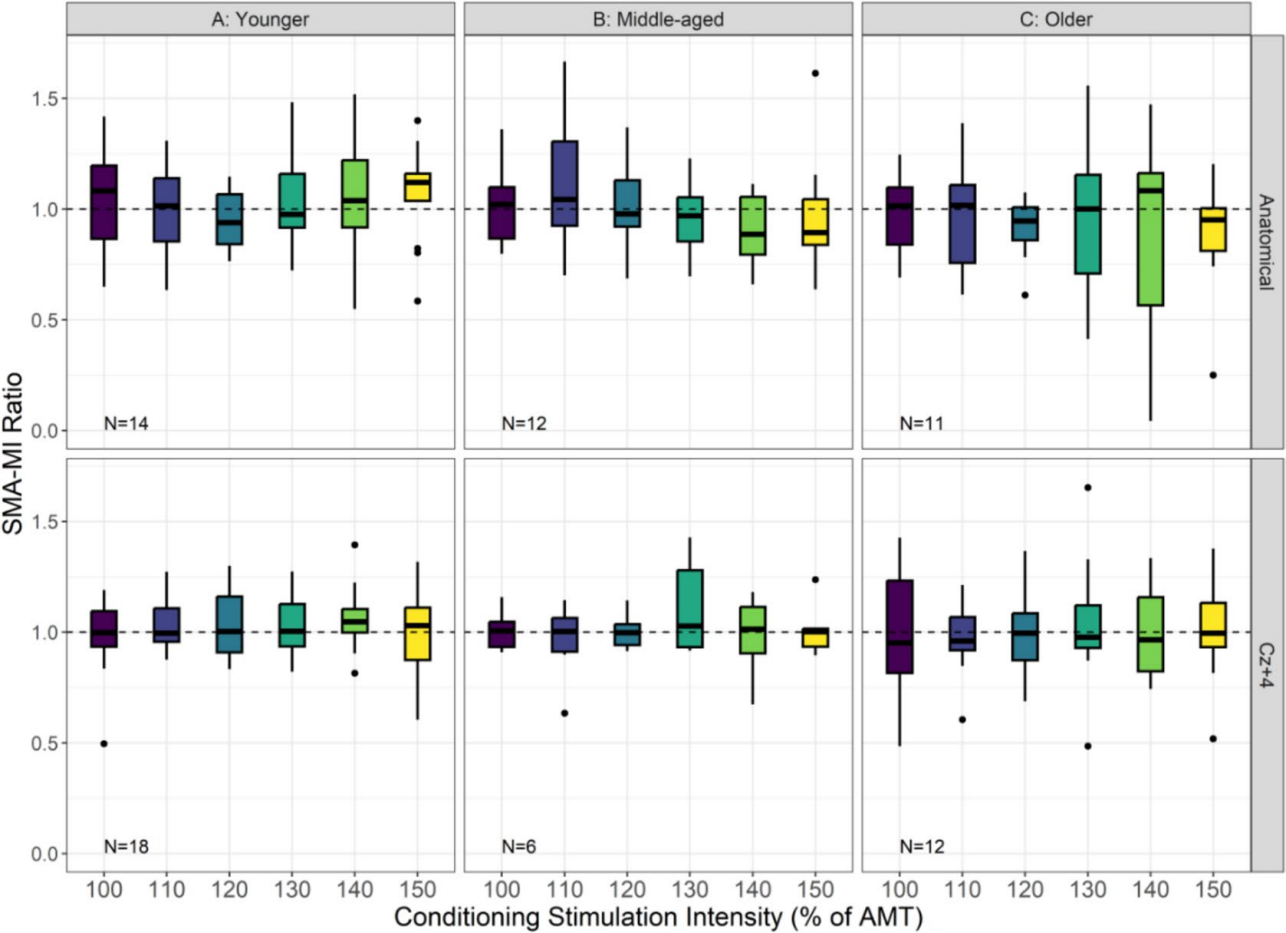
Panel **A**, **B**, and **C** show SMA–M1 ratios (y-axis) for younger (18–39 years), middle-aged (40–59 years), and older adults (60 + years), respectively, for each of the six stimulation intensities (x-axis) at anatomical SMA (top panels) and Cz + 4 (bottom panels) stimulation sites. The lower and upper hinges represent the 25th and 75th percentiles, respectively. The whiskers extend to values that are up to 1.5 times the interquartile range (the difference between the 25th and 75th percentiles) below the lower hinge and above the upper hinge. Points that lie beyond the whiskers are shown as filled circles, indicating outliers. SMA–M1 ratios less than and greater than 1 (dashed horizontal line) represent inhibitory and facilitatory interactions, respectively. The y-axis has been cropped at 1.7 to highlight the boxplots (see supplementary materials for uncropped version)

Figure 3 shows the SMA–M1 ratios for the three age groups, at each CS intensity and SMA stimulation site. The Bayesian GLMM analysing AGE, TMS TYPE, and SITE showed an inconclusive (all pds < 91.3%; all HDI in ROPE between 5.7 and 24.9%) 3-way interaction effect, and thus the null hypothesis was not accepted or rejected. Similarly, the AGE and TMS TYPE contrasts showed inconclusive effects (all pds < 91.6%; all HDI in ROPE between 9.5% and 42.0%). Likewise, the SITE and TMS TYPE contrasts highlighted inconclusive effects (all pds < 87.7%; all HDI in ROPE between 15.2 and 53.9%). These findings do not offer clear evidence for or against a practically meaningful effect of CS intensity on M1 excitability across age groups and stimulation sites.

**Fig. 4:**
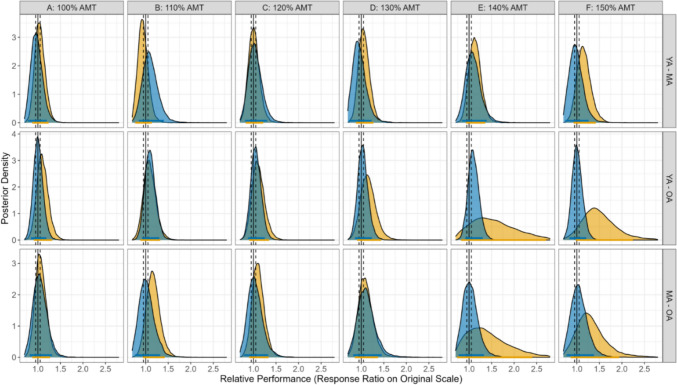
Posterior distributions of pairwise contrasts in TMS-derived ratios across stimulation conditions. The x-axis represents ratios on the original scale, where 1.0 (solid black line) indicates no group difference; the y-axis reflects the relative likelihood of each value. Vertical dashed lines indicate the null value and the Region of Practical Equivalence (ROPE: 0.95–1.05). Coloured bars show the 89% Highest Density Intervals (HDIs); when the HDI overlaps the ROPE, the group difference is considered inconclusive. Blue bars represent stimulation at the Cz + 4 site, and yellow bars represent stimulation at the anatomical SMA site. YA, MA, and OA refer to younger, middle-aged, and older adults, respectively

Figure 4 shows the posterior distributions of all pairwise group contrasts for SMA–M1 ratios at each CS intensity.

### Associations between SMA–M1 connectivity and assembly pegboard performance

Bayesian Spearman correlation analyses revealed weak and uncertain associations between pegboard assembly scores and SMA–M1 connectivity across age groups. At 140% RMT, all posterior medians were small (|ρ|≤ 0.15), with 29–43% of the posterior mass falling within the ROPE (± 0.10), suggesting negligible and inconclusive effects. At 150% RMT, the middle-aged group showed a potentially moderate negative correlation (ρ =  − 0.30, 14% in ROPE), but the evidence was not strong enough to draw firm conclusions. Overall, the pd values remained below 93%, and ROPE inclusion rates were high, supporting the absence of a clear relationship in any group.

### MEP amplitude variability

Bayesian comparisons of MEP variability showed that stimulation at the anatomical site produced meaningfully greater variability than Cz + 4 in younger adults (54.7% higher (95% CI: 17 – 104%); pd = 99.9%; ROPE = 0%). However, the effect was inconclusive in middle-aged (pd = 97.1%; ROPE = 2.6%) and older adults (pd = 75.2%; ROPE = 20.0%). For age-related comparisons at the anatomical site, older adults showed meaningfully lower variability than younger adults (48.7% lower (95% CI: 8 – 105%); pd = 99.2%; ROPE = 0%). However, the comparisons between younger and middle-aged adults (pd = 83.3%; ROPE = 15.6%) and between middle-aged and older adults (pd = 93.4%; ROPE = 8.7%) were inconclusive. At the Cz + 4 site, all age-related contrasts were also inconclusive, with pd values ≤ 95.2% and ROPE inclusion above 5%. Bayesian Spearman correlation analyses showed no evidence of a relationship between MEP variability (CV) and SMA–M1 connectivity across all participants. At the anatomical site, the posterior median was near zero (ρ =  − 0.01), with 52% of the posterior distribution within the ROPE (± 0.10), indicating an inconclusive effect. At the Cz + 4 site, the correlation was also weak (ρ = 0.08), with 45% of the posterior mass in the ROPE. The pd values remained below 70%, further reinforcing the absence of a meaningful association between trial-to-trial variability and connectivity estimates.

## Discussion

We investigated the effects of CS intensity and age group on connectivity between SMA and M1, and possible associations between SMA–M1 connectivity and bilateral performance. We found (1) no conclusive evidence for age-related differences in SMA–M1 connectivity; (2) no conclusive evidence that suprathreshold CS intensity affects SMA–M1 connectivity; (3) no significant relationship between SMA–M1 connectivity and bilateral performance.

### No conclusive age-related differences in SMA–M1 connectivity

**Fig. 5:**
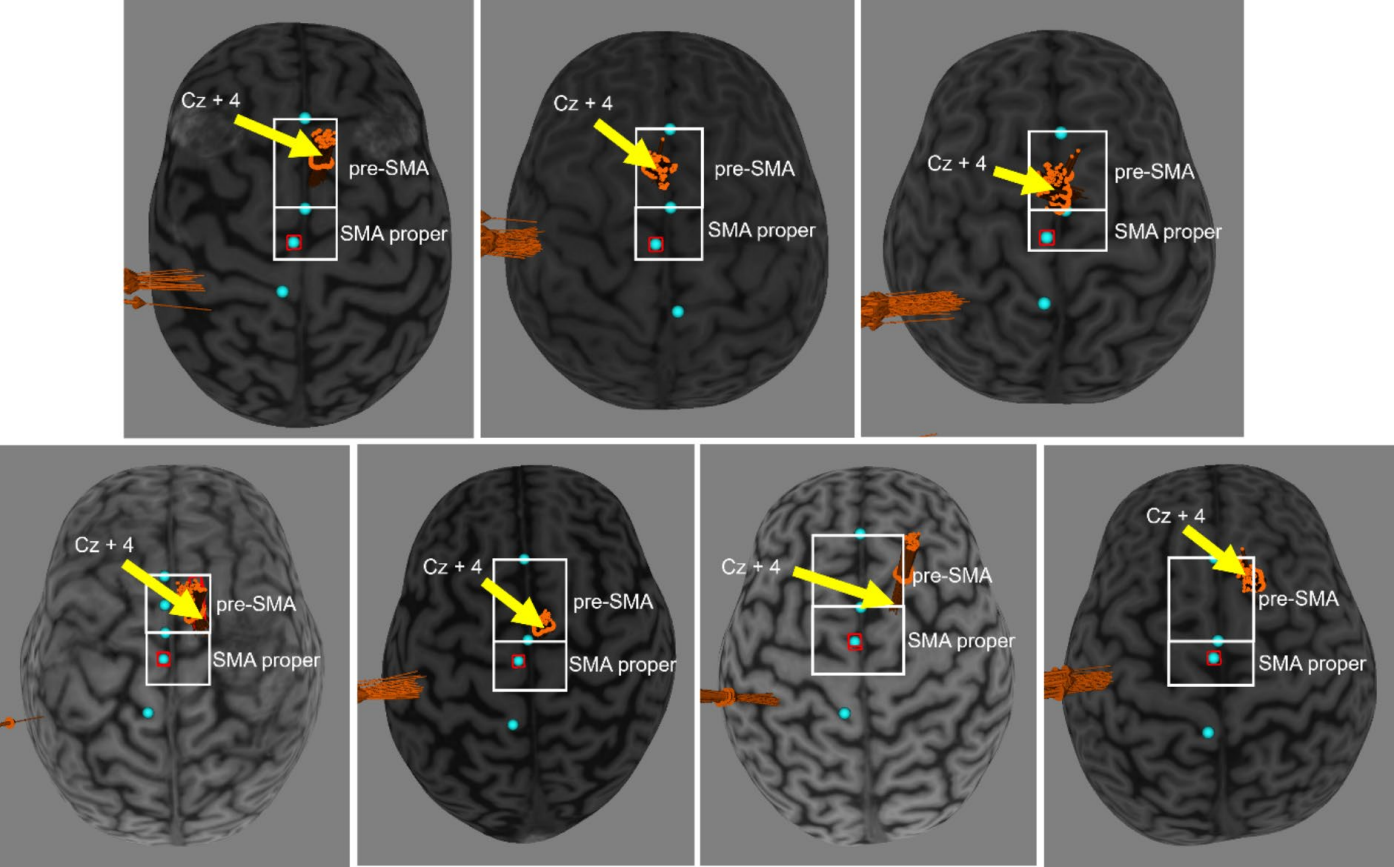
Individual imaging scans for participants whose Cz + 4 locations were situated over the pre-SMA instead of SMA-proper

We found no conclusive differences in SMA–M1 connectivity between younger, middle-aged, and older adults. This is inconsistent with previous research that found significantly weaker SMA–M1 connectivity in older adults than younger adults (Green et al. 2018; Rurak et al. 2021b). The lack of conclusive SMA–M1 facilitation in younger adults was also contrary to previous studies (Arai et al. 2011, 2012; Green et al. 2018; Rurak et al. 2021b). The inconsistency of our findings with previous studies could be due to the choice of stimulation site for SMA. In the absence of imaging data, many studies investigating SMA–M1 connectivity have used Cz + 4 as the SMA stimulation site (Arai et al. 2011, 2012; Green et al. 2018; Rurak et al. 2021a, 2021a, 2021b, 2024). This was based on previous studies that verified the Cz + 4 localisation of SMA using MRI-navigation in seven (Arai et al. 2012) and five (Arai et al. 2011) participants. Through a post-study examination of individual imaging scans in the current study, we found that Cz + 4 was potentially situated over the pre-supplementary motor area (pre-SMA) instead of SMA-proper for seven participants (see Fig. 5). There are key differences between SMA-proper and pre-SMA. In terms of connections with M1, SMA-proper has direct connections to M1 (Dum and Strick 2002; Luppino et al. 1993) while pre-SMA is only sparsely connected to M1 (Dum and Strick 2005; Tokuno and Tanji 1993). These differences in connectivity between SMA-proper and pre-SMA and M1 are consistent with a previous dual-site TMS study that found significantly smaller MEP amplitudes when a CS was delivered to pre-SMA compared to SMA-proper (Arai et al. 2011). The use of Cz + 4 in previous studies might have resulted in more variable stimulation of pre-SMA and –-proper across individuals, and might explain, in part, the inconsistencies in findings between studies.

The absence of age-related differences in SMA–M1 connectivity in the current study is inconsistent with previous research that have found TMS-based connectivity between SMA and M1 to be weaker for older adults compared with younger adults (Green et al. 2018; Rurak et al. 2021b). In contrast to these findings, other studies have found age-related *increases* in functional magnetic resonance imaging (fMRI)-based connectivity between SMA and M1 (Michely et al. 2018; Solesio-Jofre et al. 2014). Notwithstanding the different processes measured using TMS- and fMRI-based measures of connectivity, this suggests complex changes in SMA–M1 connectivity with advancing age. Indeed, age-related increases in connectivity between motor regions might reflect ineffective compensatory mechanisms that are associated with poor motor performance (Loibl et al. 2011).

The lack of conclusive SMA–M1 facilitation in younger adults could also be due to SMA exerting inhibitory effects on M1: non-human primate studies found that intracortical microstimulation of SMA can both inhibit or facilitate M1 activity, depending on the neuronal subpopulations within SMA that are stimulated (Côté et al. 2020; Tokuno and Nambu 2000). Inhibitory effects from the intracortical microstimulation of SMA were stronger with longer ISIs of 6 and 10 ms, compared with shorter ISIs of 1 and 2 ms (Côté et al. 2020). An ISI of 7 ms was used for the current study as it has highest test–retest reliability when compared with ISIs of 6 and 8 ms (Rurak et al. 2021a) – it is possible that the stronger inhibitory effects at this ISI might have dominated facilitatory effects. Moreover, the modulatory effects from SMA on M1 were found to be more subtle compared to other premotor regions such as the ventral and dorsal premotor cortex (Côté et al. 2020). This might account for the lack of conclusive SMA–M1 facilitation in younger adults.

The lack of significant SMA–M1 facilitation in younger adults, and lack of conclusive age group and CS intensity-specific differences, could also be due to the inter-individual variability of SMA–M1 connectivity (as shown by the error bars in Fig. 3). The inter-individual variability of cortico-cortical connectivity has been previously reported for SMA–M1, S1–M1, bilateral M1, and between the dorsolateral prefrontal cortex and M1 (M. J. N. Brown et al. 2019; Ferbert et al. 1992; Rurak et al. 2021b; Wang et al. 2020). A recent paper has found that variability in SMA–M1 connectivity might be due to individual differences in cortical volume, with greater SMA–M1 facilitation in younger adults with larger SMA volumes (Neige et al. 2023). It would be interesting for future research to determine whether the relationship between SMA–M1 connectivity and SMA volume is evident in older adults, and whether the nature of this relationship changes across the lifespan. In the current study, the results from the CV analysis on SI_1mV_-alone trials showed greater variability in SI_1mV_-alone trials at the anatomical SMA site compared to the Cz + 4 site, with greater variability in younger than older participants at the anatomical SMA site. It is not clear what underlies the greater variability at the anatomical SMA site than the Cz + 4 site, however, given that we found no significant correlation between SMA–M1 connectivity and CV of SI_1mV-_alone trials, the variability of SMA–M1 connectivity is unlikely due to variability in corticospinal excitability alone.

It is also possible that the lack of conclusive age-related differences was due to the middle-aged and older adults in the current sample being high-functioning and having ‘young-like’ SMA volumes. Future studies should also investigate if M1 cortical volume affects SMA–M1 connectivity, as lower M1 cortical thickness has been associated with lower RMT (less cortical excitability) (List et al. 2013). Similarly, the effects of SMA–M1 structural (anatomical) connectivity on SMA–M1 connectivity should be explored; greater structural connectivity between bilateral M1 was previously found to be associated with stronger interhemispheric M1 inhibition (Fujiyama et al. 2016).

Finally, the inconsistency of our findings with previous studies could be due to differences in statistical approach: in the current study, a GLMM was performed using trial-level data instead of *t* tests and analyses of variance (ANOVAs) on aggregate data (i.e., average MEP amplitudes for each participant). As a point of illustration, Rurak et al. (2021b) found significant SMA–M1 facilitation in younger adults as one-sample *t* tests showed SMA–M1 connectivity ratios were significantly greater than 1.0. However, analysis of the same data using a GLMM on trial-level data MEP amplitudes found no significant differences between SI_1mV_-alone and dual-site trials, indicating no significant SMA–M1 facilitation in the same younger adults (Rurak et al. 2024). Trial-level analyses also found no significant differences in SI_1mV_-alone and dual-site trials between younger and older adults, indicating no age-related differences in SMA–M1 connectivity (Rurak et al. 2024).

### No conclusive effects of CS intensity on SMA–M1 connectivity

The inconclusive differences in SMA–M1 connectivity across six suprathreshold CS intensities suggest that the interaction between these sites might be independent of conditioning intensity. While this is the first study to comprehensively examine the effect of CS intensity on SMA–M1 connectivity, previous research has shown no facilitation between SMA and M1 when the CS intensity was subthreshold, but facilitation between SMA and M1 when the CS intensity was 140% AMT (Arai et al. 2011, 2012). Targeting connectivity between other brain regions, previous research found significant facilitation between M1 and posterior parietal cortex when CS intensity was 90% resting motor threshold (RMT) and not 70%, 110%, or 130% RMT (Koch et al. 2007), and greater inhibition between M1 and primary somatosensory cortex at CS intensities of 140% and 160% AMT compared with 50–100% AMT (M. J. N. Brown et al. 2019). It is unclear why we found no conclusive consistent and significant evidence of CS intensity on SMA–M1 connectivity. It is possible that optimal SMA–M1 facilitation occurs at CS intensities beyond the range that was tested in this study, i.e., below 100% AMT or above 150% AMT. However, it is worth noting that current TMS machine constraints make it practically difficult to measure intensities above 150% AMT; in our study, 10 participants had 150% AMT intensities that exceeded MSO, and 8 participants had 140% AMT intensities that exceeded MSO.

### SMA stimulation site

There were no consistent differences in SMA–M1 connectivity between the two methods used to determine SMA stimulation site, specifically, the anatomical SMA stimulation location and the standardised Cz + 4 stimulation location. Notably, MEP amplitudes for SI_1mV_-alone trials were more variable for anatomical SMA than Cz + 4. This could be due to the anatomical SMA site being closer to M1 than Cz + 4, and the positioning of the coil at anatomical SMA might have affected the positioning of the coil targeting M1. In the current study, the stimulation of pre-SMA instead of SMA-proper in some participants might have contributed to the inconclusive effects of CS intensity and age on SMA–M1 connectivity. When the analysis was limited to participants confirmed to have SMA-proper stimulation, the findings were largely unchanged. There was inconclusive evidence that age, stimulation intensity, or stimulation site meaningfully influenced how SMA affected M1 (see Supplementary Materials for full analysis code). This suggests that even when targeting was verified, the effects of SMA stimulation on M1 excitability remained unclear.

### Motor functioning

Motor functioning was measured using the Purdue Pegboard task, and older participants had significantly poorer motor performance than younger and middle-aged participants on all subtests (Fig. 1). This is in line with age-related motor declines (Krehbiel et al. 2017). Notably, the median scores of younger and middle-aged participants on all subtests suggests that middle-aged individuals might be more young-like than old-like in manual dexterity and bimanual coordination. White matter integrity, a measure of structural changes in the brain, has been shown to start declining around 40 years of age, with accelerated decline during old age (Beck et al. 2021). Future research will need to further investigate how these structural changes potentially underlie differential age-related changes in motor functioning.

There was no conclusive evidence supporting significant associations between SMA–M1 connectivity and motor functioning for any age groups or CS intensities. This is inconsistent with previous research, which found greater SMA–M1 facilitation (measured using CS intensity 140% AMT) to be significantly associated with better assembly subtest performance in older adults (Rurak et al. 2021b). The inter-individual variability of SMA–M1 connectivity might have contributed to the lack of significant correlations. It is also possible that the Purdue pegboard task might not be optimal for measuring bilateral functioning that is directly influenced by SMA–M1 connectivity: a previous functional MRI study found that whole hand movements resulted in stronger and more reliable blood-oxygen-level-dependent signal changes in SMA and M1 than fine finger movements (Grefkes et al. 2008). As the Purdue pegboard task involves both fine and gross movements, rather than just fine movements, this might have contributed to the lack of conclusive association between SMA–M1 connectivity and Purdue pegboard task performance. Furthermore, the Purdue pegboard task might not be adequately sensitive to smaller differences in bilateral performance. For instance, an individual who just manages to insert 15 pegs will get the same score as someone who almost manages to insert the 16th peg within the allocated 30 s. A more sensitive measure might to use a marker-less tracking software (e.g., Mathis et al. 2018) to track and measure hand motions in a video-recording of the task performance. The stimulation of pre-SMA instead of SMA-proper for some participants might have also affected the measurement of SMA–M1 connectivity and its association with task performance. Functionally, SMA-proper is generally implicated in the initiation of voluntary movements and pre-SMA is generally implicated in the planning, coordination, and inhibition of motor responses (Coull et al. 2016; Nakajima et al. 2022). Therefore, SMA-proper would likely play a larger role in the unimanual and simple bimanual subtests of the Purdue Pegboard, while pre-SMA would be more involved in the motor coordination required for the assembly subtest. The stimulation of pre-SMA instead of SMA-proper for some participants might have contributed to the lack of conclusive correlations between SMA–M1 connectivity and task performance.

Finally, previous research has found that large-scale inter-network connectivity (e.g., between motor and non-motor networks) play a larger role than intra-network connectivity (e.g., between SMA and M1 in the motor network) in age-related declines in motor functioning (King et al. 2018). This might account for the lack of conclusive correlations between SMA–M1 connectivity and motor task performance in older participants in the current study. Furthermore, previous research has found age-related increases in the recruitment of non-motor regions such as the prefrontal cortex to compensate for a less efficient motor network (Michely et al. 2018). Therefore, connectivity between motor regions and the prefrontal cortex might play a larger role in motor performance than SMA–M1 connectivity in older adults.

## Limitations and future directions

The current sample was biased against individuals with high motor thresholds: individuals for whom 140% AMT exceeded MSO were excluded. Current TMS machine constraints make it practically difficult to administer stimulation at higher intensities. There were also challenges in recruiting middle-aged participants, potentially due to higher work and family commitments in this age group (Alburez-Gutierrez et al. 2021); our study might have been underpowered by the relatively smaller sample of middle-aged participants. The potential stimulation of the pre-SMA instead of SMA-proper for a few participants is also a key limitation of this study. While it is ideal for future research to use individual scans for SMA localisation, this is costly and not practical. Other methods of localising SMA include 15% of the distance between nasion and inion, anterior to Cz (Hawken et al. 2016), applying TMS to the Cz and moving the coil anteriorly until MEPs are not elicited in the tibialis anterior muscle (Carlsen et al. 2015), 1 cm anterior to the abductor hallucis hotspot (Raux et al. 2010), 2–4 cm anterior to Cz where no MEPs can be elicited in the shoulder, trunk, or lower limb muscles (Oliveri et al. 2003), and 3 cm anterior to the tibialis anterior hotspot (Shirota et al. 2012). Given the lack of consensus regarding SMA localisation, a future large-scale study is needed to determine the optimal stimulation site for SMA when imaging scans are not viable.

Future research should examine both intra- and inter-session reliability across a range of conditioning stimulus intensities. This would provide a more comprehensive understanding of the stability and robustness of connectivity measures and improve the generalisability of findings across different experimental protocols and potential clinical populations. It also remains unclear whether administering the pegboard tests in a fixed order may have introduced short-term learning or fatigue effects that differed between older and younger adults, potentially influencing the relationship between SMA–M1 connectivity and bilateral performance. Younger adults may also exhibit faster learning rates, meaning that performance on later tasks could be more affected by prior task exposure. To minimise these potential bias, future studies should consider randomising the order of pegboard tasks. Addressing both issues would enhance the interpretability and translational relevance of the findings.

## Conclusion

This is the first study to investigate SMA–M1 connectivity and bilateral functioning in younger, middle-aged, and older participants. Contrary to our hypotheses, there were no conclusive differences in SMA–M1 connectivity between CS intensities and age groups, and no conclusive associations between SMA–M1 connectivity and bilateral performance. For insight into the inter-individual variability of SMA–M1 connectivity, future studies should also examine the influence of SMA and M1 morphometry, and the structural connectivity between these regions. A systematic and large-scale investigation into the optimal localisation of SMA is also warranted.

## Supplementary Information

Below is the link to the electronic supplementary material.

Supplementary file1 (DOCX 217 KB)

## Data Availability

The datasets generated during and/or analysed during the current study are available from the corresponding author on reasonable request.

## Abbreviations

AMT: Active motor threshold
CS: Conditioning stimulus
CV: Coefficient of variation
EMG: Electromyography
FDI: First dorsal interosseous
GLMM: Generalised linear mixed model
HDI: Highest density interval
ISI: Interstimulus intervals
MEP: Motor evoked potential
MRI: Magnetic resonance imaging
MSO: Maximum stimulator output
M1: Primary motor cortex
NMDA: N-methyl-D-aspartate
pd: Probability of direction
pre-SMA: Pre-supplementary motor area
RMS: Root mean square
RMT: Resting motor threshold
ROPE: Region of practical equivalence
SMA: Supplementary motor area
TMS: Transcranial magnetic stimulation
TS: Test stimulus
