# Supplementary material for: Investigating the effects of age and conditioning stimulation intensity on SMA–M1 connectivity in younger, middle-aged, and older adults
Source: Eur J Appl Physiol. 2025 Jul 21;126(1):359–73. doi: 10.1007/s00421-025-05904-0 (PMC12881103; doi:10.1007/s00421-025-05904-0)
Supplement: Supplementary file 1 — Supplementary file1 (DOCX 217 KB) [file 421_2025_5904_MOESM1_ESM.docx]

# Supplementary Materal

## AC-PC Line

The anterior commissure (AC) and posterior commissure (PC) were defined in native subject space by manually identifying the landmarks on each participant’s T1-weighted MRI. A straight line was then positioned through the AC and PC to generate the AC–PC line. Landmarks were identified by visually inspecting the mid-sagittal slice, with the AC located as a compact white matter bundle at the anterior wall of the third ventricle, and the PC as a distinct tract at the dorsal midbrain, just above the cerebral aqueduct (see Fig. S1)


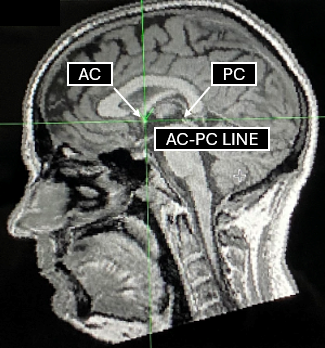


**Fig. S1** Representative T1-weighted MRI showing the anatomical landmarks used to define the anterior–posterior commissure (AC–PC) line. The anterior commissure (AC) and posterior commissure (PC) are highlighted, with a straight line connecting the two points representing the AC–PC line. These landmarks were manually identified in native subject space.

## R Code—SMAM1.R

# TMS Dictionary
# GROUP = AGE; STATE = TMS TYPE

# Loading packages ####

library(here)
library(tidyverse)
library(skimr)
library(brms)
library(emmeans)
library(bayestestR)
library(insight)
library(Rmisc)

# Fitted models ####

tms_model <- readRDS("smam1_tms_model.rds")
filtered_tms_model <- readRDS("smam1_filtered_tms_model.rds")
mt_model_beta <- readRDS("smam1_mt_beta_model.rds")
peg_model_poisson_fixed <- readRDS("smam1_peg_model.rds")
cv_model <- readRDS("smam1_cv_model.rds")
average_results <- readRDS("smam1_aggregate_analysis_results.rds")

# TMS Models

# Full sample
tms_model <- brm(MEP ~ STATE * GROUP * SITE + (STATE|gr(ID, by = GROUPSITE)),
 family = Gamma(link = "log"),
 chains = 4,
 iter = 6000,
 warmup = 1500,
 cores = 4,
 data = tms)

# SMA proper subset
filtered_tms_model <- brm(MEP ~ STATE * GROUP * SITE + (STATE|gr(ID, by = GROUPSITE)),
 family = Gamma(link = "log"),
 chains = 4,
 iter = 6000,
 warmup = 1500,
 cores = 4,
 data = filtered_tms)

# Diagnostics
summary(tms_model)
plot(tms_model)
pp_check(tms_model)

summary(filtered_tms_model)
plot(filtered_tms_model)
pp_check(filtered_tms_model)

# Full sample ####

# GROUP effects

# Contrasts
emm_group_full <- emmeans(tms_model, "trt.vs.ctrl" ~ STATE, ref = "SP", type = "response", level = 0.89)

pd(get_parameters(emm_group_full$contrasts)-1)

print(describe_posterior(emm_group_full$contrasts, rope_range = c(0.95, 1.05)), digits = 5)

# STATE * GROUP * SITE interaction

# Model ratio values
emmeans(tms_model, trt.vs.ctrl ~ STATE | GROUP | SITE, ref = "SP", type = "response", level = 0.89)$contrasts

# Contrasts
emm_state_group_site_full <- emmeans(tms_model, ~ STATE * GROUP * SITE, type = "response", level = 0.89)

cont_emm_state_group_site_full <- contrast(emm_state_group_site_full, interaction = c("trt.vs.ctrl", "pairwise", "pairwise"),
 ref = "SP")

pd(get_parameters(cont_emm_state_group_site_full)-1)

print(describe_posterior(cont_emm_state_group_site_full, rope_range = c(0.95, 1.05)), digits = 5)

# STATE * GROUP interaction

# Model ratios
emmeans(tms_model, trt.vs.ctrl ~ STATE | GROUP , ref = "SP", type = "response", level = 0.89)$contrasts

# Contrasts
emm_state_group_full <- emmeans(tms_model, ~ STATE * GROUP, type = "response", level = 0.89)

cont_emm_state_group_full <- contrast(emm_state_group_full, interaction = c("trt.vs.ctrl", "pairwise"),
 ref = "SP")

pd(get_parameters(cont_emm_state_group_full)-1)

print(describe_posterior(cont_emm_state_group_full, rope_range = c(0.95, 1.05)), digits = 5)

# STATE * SITE interaction

# Model ratios
emmeans(tms_model, trt.vs.ctrl ~ STATE | SITE, ref = "SP", type = "response", level = 0.89)$contrasts

# Contrasts
emm_state_site_full <- emmeans(tms_model, ~ STATE * SITE, type = "response", level = 0.89)

cont_emm_state_site_full <- contrast(emm_state_site_full, interaction = c("trt.vs.ctrl", "trt.vs.ctrl"),
 ref = c("SP", "Cz"))

pd(get_parameters(cont_emm_state_site_full)-1)

print(describe_posterior(cont_emm_state_site_full, rope_range = c(0.95, 1.05)), digits = 5)

# Single-pulse (SP) analysis

# Model values
emmeans(tms_model, ~ GROUP * SITE, at = list(STATE = "SP"), type = "response", level = 0.89)

# Contrasts
emm_sp_interaction_full <- emmeans(
 tms_model,
 ~ GROUP * SITE,
 at = list(STATE = "SP"),
 type = "response",
 level = 0.89
)

interaction_contrasts_full <- contrast(
 emm_sp_interaction_full,
 interaction = "pairwise",
 by = NULL,
 adjust = "holm"
)

pd(get_parameters(interaction_contrasts_full) - 1)

describe_posterior(interaction_contrasts_full, rope_range = c(0.95, 1.05), digits = 5)

# SP - GROUP

# Model values
emmeans(tms_model, ~ GROUP, at = list(STATE = "SP"), type = "response", level = 0.89)

# Contrasts
emm_sp_group <- emmeans(
 tms_model,
 ~ GROUP,
 at = list(STATE = "SP"),
 type = "response"
)

cont_emm_sp_group <- contrast(
 emm_sp_group,
 method = "pairwise",
 adjust = "holm"
)

pd(get_parameters(cont_emm_sp_group) - 1)

print(
 describe_posterior(cont_emm_sp_group, rope_range = c(0.95, 1.05)),
 digits = 5
)

# SP - SITE

# Model values
emmeans(tms_model, ~ SITE, at = list(STATE = "SP"), type = "response", level = 0.89)

# Contrasts
emm_sp_site <- emmeans(
 tms_model,
 ~ SITE,
 at = list(STATE = "SP"), # Only include SP condition
 type = "response"
)

cont_emm_sp_site <- contrast(
 emm_sp_site,
 method = "pairwise",
 adjust = "holm"
)

pd(get_parameters(cont_emm_sp_site) - 1)

print(
 describe_posterior(cont_emm_sp_site, rope_range = c(0.95, 1.05)),
 digits = 5
)


# Filtered model ####

# GROUP effects

# Contrasts
emm_group_fil <- emmeans(filtered_tms_model, "trt.vs.ctrl" ~ STATE, ref = "SP", type = "response", level = 0.89)

pd(get_parameters(emm_group_fil$contrasts)-1)

print(describe_posterior(emm_group_fil$contrasts, rope_range = c(0.95, 1.05)), digits = 5)

# STATE * GROUP * SITE interaction

# Model ratio values
emmeans(filtered_tms_model, trt.vs.ctrl ~ STATE | GROUP | SITE, ref = "SP", type = "response", level = 0.89)

# Contrasts
emm_state_group_site_fil <- emmeans(filtered_tms_model, ~ STATE * GROUP * SITE, type = "response", level = 0.89)

cont_emm_state_group_site_fil <- contrast(emm_state_group_site_fil, interaction = c("trt.vs.ctrl", "pairwise", "pairwise"),
 ref = "SP")

pd(get_parameters(cont_emm_state_group_site_fil)-1)

print(describe_posterior(cont_emm_state_group_site_fil, rope_range = c(0.95, 1.05)), digits = 5)

# STATE * GROUP interaction

# Model ratios
emmeans(filtered_tms_model, trt.vs.ctrl ~ STATE | GROUP , ref = "SP", type = "response", level = 0.89)

# Contrasts
emm_state_group_fil <- emmeans(filtered_tms_model, ~ STATE * GROUP, type = "response", level = 0.89)

cont_emm_state_group_fil <- contrast(emm_state_group_fil, interaction = c("trt.vs.ctrl", "pairwise"),
 ref = "SP")

pd(get_parameters(cont_emm_state_group_fil)-1)

print(describe_posterior(cont_emm_state_group_fil, rope_range = c(0.95, 1.05)), digits = 5)

# STATE * SITE interaction

# Model ratios
emmeans(filtered_tms_model, trt.vs.ctrl ~ STATE | SITE, ref = "SP", type = "response", level = 0.89)

# Contrasts
emm_state_site_fil <- emmeans(filtered_tms_model, ~ STATE * SITE, type = "response", level = 0.89)

cont_emm_state_site_fil <- contrast(emm_state_site_fil, interaction = c("trt.vs.ctrl", "trt.vs.ctrl"),
 ref = c("SP", "Cz"))

pd(get_parameters(cont_emm_state_site_fil)-1)

print(describe_posterior(cont_emm_state_site_fil, rope_range = c(0.95, 1.05)), digits = 5)

# Single-pulse (SP) analysis

# Model values
emmeans(filtered_tms_model, ~ GROUP * SITE, at = list(STATE = "SP"), type = "response", level = 0.89)

# Contrasts
emm_sp_interaction_fil <- emmeans(
 filtered_tms_model,
 ~ GROUP * SITE,
 at = list(STATE = "SP"),
 type = "response",
 level = 0.89
)

interaction_contrasts_fil <- contrast(
 emm_sp_interaction_fil,
 interaction = "pairwise",
 by = NULL,
 adjust = "holm"
)

pd(get_parameters(interaction_contrasts_fil) - 1)

describe_posterior(interaction_contrasts_fil, rope_range = c(0.95, 1.05), digits = 5)

# SP - GROUP

# Model values
emmeans(filtered_tms_model, ~ GROUP, at = list(STATE = "SP"), type = "response", level = 0.89)

# Contrasts
emm_sp_group_fil <- emmeans(
 filtered_tms_model,
 ~ GROUP,
 at = list(STATE = "SP"),
 type = "response"
)

cont_emm_sp_group_fil <- contrast(
 emm_sp_group_fil,
 method = "pairwise",
 adjust = "holm"
)

pd(get_parameters(cont_emm_sp_group_fil) - 1)

print(
 describe_posterior(cont_emm_sp_group_fil, rope_range = c(0.95, 1.05)),
 digits = 5
)

# SP - SITE

# Model values
emmeans(filtered_tms_model, ~ SITE, at = list(STATE = "SP"), type = "response", level = 0.89)

# Contrasts
emm_sp_site_fil <- emmeans(
 filtered_tms_model,
 ~ SITE,
 at = list(STATE = "SP"),
 type = "response"
)

cont_emm_sp_site_fil <- contrast(
 emm_sp_site_fil,
 method = "pairwise",
 adjust = "holm"
)

pd(get_parameters(cont_emm_sp_site_fil) - 1)

print(
 describe_posterior(cont_emm_sp_site_fil, rope_range = c(0.95, 1.05)),
 digits = 5
)


# AMT analysis ####

mt_model_beta <- brm(
 MT_prop ~ GROUP + (1 | ID),
 family = Beta(),
 data = df_mt,
 chains = 4,
 iter = 10000,
 warmup = 5000,
 cores = 4,
 control = list(adapt_delta = 0.999, max_treedepth = 20)
)

# Diagnostics
summary(mt_model_beta)
plot(mt_model_beta)
pp_check(mt_model_beta)

# Scores - Response
emmeans(mt_model_beta, pairwise ~ GROUP, type = "response", level = 0.89)

# Contrasts
emm_mt_group <- emmeans(mt_model_beta, pairwise ~ GROUP, type = "response", level = 0.89)

pd(get_parameters(emm_mt_group$contrasts)-1)

print(describe_posterior(emm_mt_group$contrasts, rope_range = c(0.95, 1.05)), digits = 5)

# Pegboard analysis ####

peg_model_poisson_fixed <- brm(
 Score ~ GROUP * Inst + (Inst|gr(ID, by = GROUP)),
 family = poisson(link = "log"),
 chains = 4,
 iter = 6000,
 warmup = 1500,
 cores = 4,
 data = peg_data
)

# Diagnostics
summary(peg_model_poisson_fixed)
plot(peg_model_poisson_fixed)
pp_check(peg_model_poisson_fixed, type = "hist")

# GROUP effect
emm_peg_group <- emmeans(peg_model_poisson_fixed, pairwise ~ GROUP, type = "response", level = 0.89)

pd(get_parameters(emm_peg_group$contrasts)-1)

print(describe_posterior(emm_peg_group$contrasts, rope_range = c(0.95, 1.05)), digits = 5)

# Scores - Rate
emmeans(peg_model_poisson_fixed, pairwise ~ GROUP | Inst, type = "response", level = 0.89)

# Contrasts - GROUP by Inst
emm_peg_group_inst <- emmeans(peg_model_poisson_fixed, pairwise ~ GROUP | Inst, type = "response", level = 0.89)

pd(get_parameters(emm_peg_group_inst$contrasts)-1)

print(describe_posterior(emm_peg_group_inst$contrasts, rope_range = c(0.95, 1.05)), digits = 5)

# CV analysis ####

cv_model <- brm(
 CV ~ GROUP * SITE + (1 | ID),
 data = cv_by_id_site,
 family = Gamma(link = "log"),
 chains = 4,
 iter = 20000,
 warmup = 8000,
 cores = 4,
 control = list(
 adapt_delta = 0.99999,
 max_treedepth = 30
 )
)

# Diagnostics
summary(cv_model_interact)
plot(cv_model)
pp_check(cv_model)

# Site contrasts within each group
emm_site_within_group <- emmeans(cv_model_interact, pairwise ~ SITE | GROUP, type = "response", level = 0.89)

site_contrasts <- emm_site_within_group$contrasts

pd(get_parameters(site_contrasts) - 1)

print(describe_posterior(site_contrasts, rope_range = c(0.95, 1.05)), digits = 5)

# Group contrasts within each site
emm_group_within_site <- emmeans(cv_model_interact, pairwise ~ GROUP | SITE, type = "response", level = 0.89)

group_contrasts <- emm_group_within_site$contrasts

pd(get_parameters(group_contrasts) - 1)

print(describe_posterior(group_contrasts, rope_range = c(0.95, 1.05)), digits = 5)

# Average-level analysis ####
library(dplyr)
library(tidyr)
library(purrr)
library(tibble)

df_avg <- tms %>%
 dplyr::filter(STATE %in% c("SP", "100", "110", "120", "130", "140", "150")) %>%
 dplyr::group_by(ID, GROUP, SITE, STATE) %>%
 dplyr::summarise(mean_MEP = mean(MEP, na.rm = TRUE), .groups = "drop") %>%
 as.data.frame()

df_ratio_100 <- df_avg %>%
 dplyr::filter(STATE %in% c("SP", "100")) %>%
 dplyr::group_by(ID, GROUP, SITE) %>%
 dplyr::summarise(ratio = mean_MEP[match("100", STATE)] / mean_MEP[match("SP", STATE)],
 STATE = "100", .groups = "drop")

df_ratio_110 <- df_avg %>%
 dplyr::filter(STATE %in% c("SP", "110")) %>%
 dplyr::group_by(ID, GROUP, SITE) %>%
 dplyr::summarise(ratio = mean_MEP[match("110", STATE)] / mean_MEP[match("SP", STATE)],
 STATE = "110", .groups = "drop")

df_ratio_120 <- df_avg %>%
 dplyr::filter(STATE %in% c("SP", "120")) %>%
 dplyr::group_by(ID, GROUP, SITE) %>%
 dplyr::summarise(ratio = mean_MEP[match("120", STATE)] / mean_MEP[match("SP", STATE)],
 STATE = "120", .groups = "drop")

df_ratio_130 <- df_avg %>%
 dplyr::filter(STATE %in% c("SP", "130")) %>%
 dplyr::group_by(ID, GROUP, SITE) %>%
 dplyr::summarise(ratio = mean_MEP[match("130", STATE)] / mean_MEP[match("SP", STATE)],
 STATE = "130", .groups = "drop")

df_ratio_140 <- df_avg %>%
 dplyr::filter(STATE %in% c("SP", "140")) %>%
 dplyr::group_by(ID, GROUP, SITE) %>%
 dplyr::summarise(ratio = mean_MEP[match("140", STATE)] / mean_MEP[match("SP", STATE)],
 STATE = "140", .groups = "drop")

df_ratio_150 <- df_avg %>%
 dplyr::filter(STATE %in% c("SP", "150")) %>%
 dplyr::group_by(ID, GROUP, SITE) %>%
 dplyr::summarise(ratio = mean_MEP[match("150", STATE)] / mean_MEP[match("SP", STATE)],
 STATE = "150", .groups = "drop")

df_all_ratios <- dplyr::bind_rows(
 df_ratio_100,
 df_ratio_110,
 df_ratio_120,
 df_ratio_130,
 df_ratio_140,
 df_ratio_150
)

group_levels <- levels(df_all_ratios$GROUP)
group_pairs <- combn(group_levels, 2, simplify = FALSE)
state_levels <- levels(df_all_ratios$STATE)
site_levels <- unique(df_all_ratios$SITE)

get_group_contrast <- function(state, site, g1, g2) {
 df_sub <- df_all_ratios %>%
 filter(STATE == state, SITE == site, GROUP %in% c(g1, g2))

 g1_vals <- df_sub %>% filter(GROUP == g1) %>% pull(log10_ratio)
 g2_vals <- df_sub %>% filter(GROUP == g2) %>% pull(log10_ratio)

 median_diff <- median(g2_vals) - median(g1_vals)
 fold_change <- 10^median_diff
 p_value <- wilcox.test(g2_vals, g1_vals, exact = FALSE)$p.value

 tibble(
 STATE = state,
 SITE = site,
 GROUP_1 = g1,
 GROUP_2 = g2,
 median_log10_diff = median_diff,
 fold_change = fold_change,
 p_value = p_value
 )
}

combo_grid <- expand_grid(
 STATE = state_levels,
 SITE = site_levels,
 PAIR = group_pairs
)

average_results <- pmap_dfr(combo_grid, function(STATE, SITE, PAIR) {
 get_group_contrast(STATE, SITE, PAIR[[1]], PAIR[[2]])
})

average_results_df <- as.data.frame(average_results)

print(average_results_df)

## Normalised MEP analysis (aggregate-level)

Mean MEPs were calculated for each participant for each TMS TYPE. Response ratios (TMS TYPE/SP) were log10-transformed. For each TMS TYPE and SITE, GROUP differences in the median log-transformed ratios were assessed using Wilcoxon rank-sum tests. Fold-changes were obtained by back-transforming the median differences. Comparisons were based on available data, and missing values for some participants at 150/SP were excluded pairwise from the relevant group comparisons, which led to NA values for fold-change estimates at this intensity only.

Across all TMS TYPE ratios and SITEs, fold-change differences between GROUPs were small (generally <10%) and inconsistent in direction. The largest observable difference occurred at anatomical SITE, 140/SP, where the middle-aged group showed a ~15% lower median ratio than the younger-aged group (fold-change = 0.85, *p* = 0.19). At 150/SP, the comparison between younger- and older-aged adults at the anatomical SITE yielded the lowest *p*-value (*p* = 0.068), although the direction and magnitude of the difference were not determined due to missing data.
